# Tris(piperazine-1,4-diium) bis­[hexa­chloridoindate(III)] tetra­hydrate

**DOI:** 10.1107/S1600536811007355

**Published:** 2011-03-09

**Authors:** Sofiane Bouacida, Ratiba Belhouas, Boubakeur Fantazi, Chaouki Boudaren, Thierry Roisnel

**Affiliations:** a Unité de Recherche de Chimie de l’Environnement et Moléculaire Structurale, CHEMS, Université Mentouri-Constantine, 25000 Algeria; b Département Sciences de la Matière, Facult des Sciences Exactes et Sciences de la Nature et de la Vie, Université Larbi Ben M’hidi, Oum El Bouaghi 04000, Algeria; c Centre de Difractométrie X, UMR 6226 CNRS Unité Sciences Chimiques de Rennes, Université de Rennes I, 263 Avenue du Général Leclerc, 35042 Rennes, France

## Abstract

The asymmetric unit of the title compound, (C_4_H_12_N_2_)_3_[InCl_6_]_2_·4H_2_O, consists of one and half independent piperazinium cations, an hexa­chloridoindate anion and two mol­ecules of water. The In^III^ ion is six-coordinated and forms a quasi-regular octa­hedral arrangement. In the crystal, alternating layers of cations and anions are arranged parallel to (10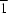) and are linked with the water mol­ecules *via* intra- and inter­molecular N—H⋯O, O—H⋯Cl, C—H⋯O and N—H⋯Cl hydrogen bonds, forming a complex three-dimensional network. Additional stabilization within the layers is provided by weak inter­molecular C—H⋯Cl inter­actions.

## Related literature

For related structures and protonated imines, see: Bouacida *et al.* (2005 ▶, 2007 ▶); Bouacida (2008 ▶); Murugavel *et al.* (2009 ▶); Polishchuk *et al.* (2009 ▶).
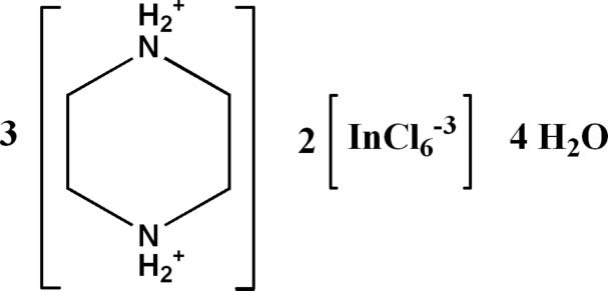


## Experimental

### 

#### Crystal data

(C_4_H_12_N_2_)_3_[InCl_6_]_2_·4H_2_O
*M* *_r_* = 991.57
Triclinic, 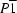

*a* = 7.9267 (3) Å
*b* = 10.0940 (3) Å
*c* = 11.8265 (5) Å
α = 89.780 (1)°
β = 89.634 (1)°
γ = 73.087 (2)°
*V* = 905.31 (6) Å^3^
*Z* = 1
Mo *K*α radiation
μ = 2.19 mm^−1^
*T* = 295 K
0.15 × 0.06 × 0.05 mm

#### Data collection

Nonius KappaCCD diffractometer
Absorption correction: multi-scan (*SADABS*; Sheldrick, 2002 ▶) *T*_min_ = 0.773, *T*_max_ = 0.938
7414 measured reflections
4131 independent reflections
3293 reflections with *I* > 2σ(*I*)
*R*_int_ = 0.024

#### Refinement

*R*[*F* ^2^ > 2σ(*F* ^2^)] = 0.030
*wR*(*F* ^2^) = 0.069
*S* = 1.09
4131 reflections
163 parameters
1 restraint
H atoms treated by a mixture of independent and constrained refinement
Δρ_max_ = 0.61 e Å^−3^
Δρ_min_ = −0.75 e Å^−3^

### 

Data collection: *COLLECT* (Hooft, 1998 ▶); cell refinement: *DENZO* (Otwinowski & Minor, 1997 ▶); data reduction: *SCALEPACK* (Otwinowski & Minor, 1997 ▶); program(s) used to solve structure: *SIR2002* (Burla *et al.*, 2003 ▶); program(s) used to refine structure: *SHELXL97* (Sheldrick, 2008 ▶); molecular graphics: *PLATON* (Spek, 2009 ▶) and *DIAMOND* (Brandenburg *et al.*, 2001 ▶); software used to prepare material for publication: *WinGX* (Farrugia, 1999 ▶).

## Supplementary Material

Crystal structure: contains datablocks global, I. DOI: 10.1107/S1600536811007355/pv2386sup1.cif

Structure factors: contains datablocks I. DOI: 10.1107/S1600536811007355/pv2386Isup2.hkl

Additional supplementary materials:  crystallographic information; 3D view; checkCIF report

## Figures and Tables

**Table 1:** Hydrogen-bond geometry (Å, °)

*D*—H⋯*A*	*D*—H	H⋯*A*	*D*⋯*A*	*D*—H⋯*A*
O1*W*—H11*W*⋯Cl2	0.84 (5)	2.43 (5)	3.248 (3)	167 (4)
O2*W*—H21*W*⋯Cl6^i^	0.80 (5)	2.58 (5)	3.353 (3)	163 (5)
O2*W*—H22*W*⋯Cl2^ii^	0.80 (6)	2.37 (6)	3.170 (3)	174 (6)
N3*A*—H31*A*⋯O1*W*^i^	0.90	1.91	2.805 (5)	178
N3*A*—H32*A*⋯O2*W*	0.90	1.95	2.843 (5)	171
N3*B*—H31*B*⋯Cl1^iii^	0.90	2.61	3.233 (3)	127
N3*B*—H31*B*⋯Cl5^iii^	0.90	2.47	3.202 (3)	138
N3*B*—H32*B*⋯Cl1	0.90	2.81	3.273 (3)	113
N3*B*—H32*B*⋯Cl3	0.90	2.37	3.231 (3)	160
N6*A*—H61*A*⋯Cl2^iv^	0.90	2.64	3.334 (3)	134
N6*A*—H61*A*⋯Cl3^iv^	0.90	2.62	3.330 (3)	136
N6*A*—H62*A*⋯Cl5^v^	0.90	2.61	3.344 (3)	140
N6*A*—H62*A*⋯Cl6^v^	0.90	2.77	3.502 (3)	139
C2*B*—H21*B*⋯O1*W*	0.97	2.47	3.306 (5)	144
C2*A*—H21*A*⋯Cl1^i^	0.97	2.72	3.470 (3)	135
C2*B*—H22*B*⋯Cl3^vi^	0.97	2.83	3.607 (3)	138
C4*A*—H41*A*⋯Cl4^v^	0.97	2.76	3.620 (3)	148
C4*A*—H42*A*⋯Cl6^ii^	0.97	2.74	3.577 (3)	145

Symmetry codes: (i) 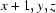; (ii) 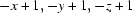; (iii) 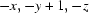; (iv) 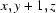; (v) 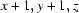; (vi) 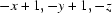.

**Fig. 1.**
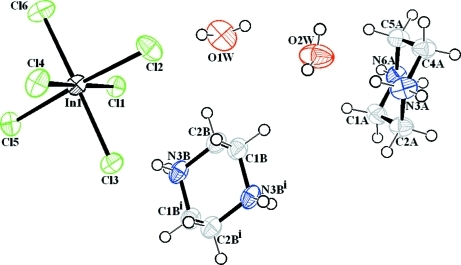
The molecular structure of (I) with the atom-labelling scheme. Displacement ellipsoids are drawn at the 30% probability level. H atoms are represented as small spheres of arbitrary radii. Symmetry code: (i) 1 - x, 1 - y, -z

**Fig. 2.**
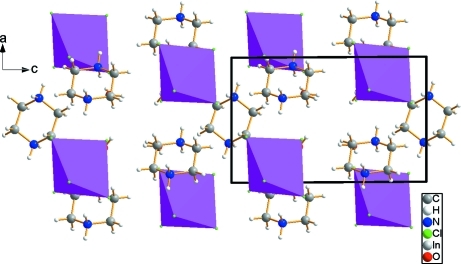
A diagram of the layered crystal packing in (I), viewed down the b axis.

**Fig. 3.**
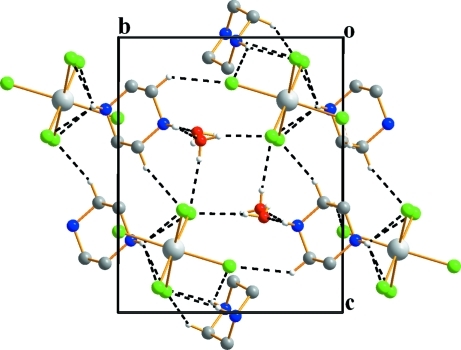
Part of the crystal structure with hydrogen bonds shown as dashed lines.

## supplementary crystallographic information

### Comment

The title compound, was prepared as part of our ongoing studies of hydrogen-bonding interactions in the crystal structures of protonated amines and imines (Bouacida, 2008; Bouacida *et al.*, 2005; 2007). We report here the synthesis and crystal structure of a new hybrid compound, (I).

The asymmetric unit of the title compound consists of one and half independent piperazinium cations, an hexachloridoindate anion and two molecules of water. The molecular structure of (I) is shown in Fig. 1. In the title compound, both imine N atoms of piperazine are protonated as in other related structures (Murugavel *et al.*, 2009; Polishchuk *et al.*, 2009). These cations adopt typical chair conformation and alternate with hexachloridoindate complex forming layers parallel to the (10–1) plane (Fig 2).

The In^III^ ion is six-coordinated and forms a quasi-regular octahedral arrangement (Fig 2). The crystal packing in (I) is governed by classical hydrogen bond, *viz* cation-anion, cation-cation, water-anion and cation-water (Table 1). In the crystal, the components of the structure are linked *via* intra and intermolecular N—H···O, O—H···Cl, C—H···O and N—H···Cl hydrogen bonds to form a complex three-dimensional network. Additional stabilization within these layers is provided by weak intermolecular C—H···Cl interactions (Fig. 3, Table 1).

### Experimental

A solution of 1 mmol InCl_3_ and 3 mmol piperazine in hydrochloric acid was slowly evaporated to dryness over a period of one week yielding colorless crystals suitable for X-ray diffraction.

### Refinement

All H atoms were visible in differnce Fourier maps but were introduced in calculated positions and treated as riding on C and N atoms with C—H = 0.97 and N—H = 0.90 Å and *U*_iso_(H) = 1.2*U*_eq_(C/N/). The positions of water H atoms were refined with *U*_iso_(H) = 1.5 *U*_eq_(O).

### Crystal data

**Table:**

(C_4_H_12_N_2_)_3_[InCl_6_]_2_·4H_2_O	*Z* = 1
*M**_r_* = 991.57	*F*(000) = 492
Triclinic, *P*1	*D*_x_ = 1.819 Mg m^−^^3^
*a* = 7.9267 (3) Å	Mo *K*α radiation, λ = 0.71073 Å
*b* = 10.0940 (3) Å	Cell parameters from 3980 reflections
*c* = 11.8265 (5) Å	θ = 2.9–27.5°
α = 89.780 (1)°	µ = 2.19 mm^−^^1^
β = 89.634 (1)°	*T* = 295 K
γ = 73.087 (2)°	Needle, colorless
*V* = 905.31 (6) Å^3^	0.15 × 0.06 × 0.05 mm

### Data collection

**Table:**

Nonius KappaCCD diffractometer	4131 independent reflections
Radiation source: Enraf Nonius FR590	3293 reflections with *I* > 2σ(*I*)
graphite	*R*_int_ = 0.024
Detector resolution: 9 pixels mm^-1^	θ_max_ = 27.5°, θ_min_ = 3.2°
CCD rotation images, thick slices scans	*h* = −8→10
Absorption correction: multi-scan (*SADABS*; Sheldrick, 2002)	*k* = −13→13
*T*_min_ = 0.773, *T*_max_ = 0.938	*l* = −15→15
7414 measured reflections	

### Refinement

**Table:**

Refinement on *F*^2^	Primary atom site location: structure-invariant direct methods
Least-squares matrix: full	Secondary atom site location: difference Fourier map
*R*[*F*^2^ > 2σ(*F*^2^)] = 0.030	Hydrogen site location: inferred from neighbouring sites
*wR*(*F*^2^) = 0.069	H atoms treated by a mixture of independent and constrained refinement
*S* = 1.09	*w* = 1/[σ^2^(*F*_o_^2^) + 0.1261*P*] where *P* = (*F*_o_^2^ + 2*F*_c_^2^)/3
4131 reflections	(Δ/σ)_max_ = 0.002
163 parameters	Δρ_max_ = 0.61 e Å^−^^3^
1 restraint	Δρ_min_ = −0.75 e Å^−^^3^

### Special details

**Table:**

Geometry. All e.s.d.'s (except the e.s.d. in the dihedral angle between two l.s. planes) are estimated using the full covariance matrix. The cell e.s.d.'s are taken into account individually in the estimation of e.s.d.'s in distances, angles and torsion angles; correlations between e.s.d.'s in cell parameters are only used when they are defined by crystal symmetry. An approximate (isotropic) treatment of cell e.s.d.'s is used for estimating e.s.d.'s involving l.s. planes.
Refinement. Refinement of *F*^2^ against ALL reflections. The weighted *R*-factor *wR* and goodness of fit *S* are based on *F*^2^, conventional *R*-factors *R* are based on *F*, with *F* set to zero for negative *F*^2^. The threshold expression of *F*^2^ > σ(*F*^2^) is used only for calculating *R*-factors(gt) *etc*. and is not relevant to the choice of reflections for refinement. *R*-factors based on *F*^2^ are statistically about twice as large as those based on *F*, and *R*- factors based on ALL data will be even larger.

### Fractional atomic coordinates and isotropic or equivalent isotropic displacement parameters (Å^2^)

**Table:**

	*x*	*y*	*z*	*U*_iso_*/*U*_eq_	
C1A	0.7212 (4)	0.9420 (3)	0.1784 (3)	0.0419 (7)	
H11A	0.7106	0.9799	0.1024	0.05*	
H12A	0.6401	0.8867	0.186	0.05*	
C1B	0.5638 (4)	0.5134 (3)	0.1119 (3)	0.04	
H11B	0.61	0.5657	0.166	0.048*	
H12B	0.5554	0.4295	0.1488	0.048*	
C2A	0.9056 (4)	0.8527 (3)	0.1970 (3)	0.0403 (7)	
H21A	0.932	0.7756	0.1446	0.048*	
H22A	0.9873	0.906	0.182	0.048*	
C2B	0.3830 (4)	0.5984 (3)	0.0742 (3)	0.0400 (7)	
H21B	0.3032	0.6173	0.1386	0.048*	
H22B	0.3895	0.6862	0.0438	0.048*	
C4A	0.8812 (4)	0.9141 (3)	0.3988 (3)	0.0415 (7)	
H41A	0.9621	0.9697	0.3916	0.05*	
H42A	0.8914	0.876	0.4747	0.05*	
C5A	0.6963 (4)	1.0037 (3)	0.3801 (3)	0.0394 (7)	
H51A	0.6144	0.9505	0.3946	0.047*	
H52A	0.6696	1.0809	0.4324	0.047*	
N3A	0.9296 (4)	0.7988 (3)	0.3150 (2)	0.0437 (6)	
H31A	1.0429	0.7492	0.325	0.052*	
H32A	0.862	0.7422	0.3266	0.052*	
N3B	0.3148 (3)	0.5233 (3)	−0.0131 (2)	0.0367 (6)	
H31B	0.2088	0.5765	−0.0365	0.044*	
H32B	0.3001	0.4455	0.0172	0.044*	
N6A	0.6748 (3)	1.0568 (3)	0.2620 (2)	0.0386 (6)	
H61A	0.5623	1.1079	0.2514	0.046*	
H62A	0.7444	1.1121	0.2506	0.046*	
Cl1	0.01218 (9)	0.49590 (7)	0.16760 (6)	0.03375 (16)	
Cl2	0.34370 (11)	0.31722 (8)	0.36015 (7)	0.0466 (2)	
Cl3	0.36503 (9)	0.21152 (7)	0.07659 (6)	0.03619 (17)	
Cl4	0.26386 (10)	0.00367 (7)	0.29365 (7)	0.04150 (18)	
Cl5	−0.06834 (10)	0.17934 (7)	0.09051 (6)	0.03826 (17)	
Cl6	−0.11484 (11)	0.28867 (8)	0.37179 (7)	0.0466 (2)	
In1	0.13014 (2)	0.247144 (19)	0.228928 (17)	0.03017 (7)	
O2W	0.7076 (5)	0.6333 (3)	0.3757 (2)	0.0694 (9)	
H21W	0.756 (7)	0.554 (5)	0.360 (4)	0.104*	
H22W	0.698 (7)	0.639 (6)	0.443 (5)	0.104*	
O1W	0.2846 (4)	0.6490 (3)	0.3457 (3)	0.073	
H11W	0.286 (6)	0.567 (5)	0.357 (4)	0.109*	
H12W	0.361 (5)	0.666 (5)	0.385 (4)	0.109*	

### Atomic displacement parameters (Å^2^)

**Table:**

	*U*^11^	*U*^22^	*U*^33^	*U*^12^	*U*^13^	*U*^23^
C1A	0.0472 (19)	0.0439 (17)	0.0357 (17)	−0.0150 (15)	−0.0066 (14)	−0.0006 (14)
C1B	0.036	0.053	0.035	−0.019	−0.005	0.006
C2A	0.0504 (19)	0.0300 (15)	0.0373 (17)	−0.0067 (14)	0.0061 (14)	−0.0019 (13)
C2B	0.0317 (16)	0.0445 (17)	0.0440 (18)	−0.0113 (14)	0.0025 (13)	0.0001 (14)
C4A	0.0402 (18)	0.0475 (18)	0.0319 (16)	−0.0050 (14)	−0.0029 (13)	0.0027 (14)
C5A	0.0399 (17)	0.0427 (17)	0.0330 (16)	−0.0081 (14)	0.0011 (13)	−0.0022 (13)
N3A	0.0460 (16)	0.0325 (13)	0.0448 (16)	0.0007 (12)	0.0051 (12)	0.0063 (11)
N3B	0.0246 (12)	0.0435 (14)	0.0424 (15)	−0.0104 (11)	−0.0045 (10)	0.0130 (12)
N6A	0.0306 (13)	0.0370 (13)	0.0434 (15)	−0.0025 (11)	−0.0039 (11)	0.0032 (11)
Cl1	0.0330 (4)	0.0259 (3)	0.0408 (4)	−0.0062 (3)	−0.0018 (3)	0.0043 (3)
Cl2	0.0442 (4)	0.0442 (4)	0.0461 (5)	−0.0041 (4)	−0.0133 (4)	−0.0085 (4)
Cl3	0.0354 (4)	0.0350 (4)	0.0368 (4)	−0.0081 (3)	0.0054 (3)	0.0028 (3)
Cl4	0.0341 (4)	0.0337 (4)	0.0540 (5)	−0.0057 (3)	−0.0029 (3)	0.0137 (3)
Cl5	0.0391 (4)	0.0343 (4)	0.0426 (4)	−0.0125 (3)	−0.0104 (3)	0.0059 (3)
Cl6	0.0439 (4)	0.0448 (4)	0.0445 (5)	−0.0026 (4)	0.0131 (3)	0.0093 (3)
In1	0.02748 (12)	0.02921 (12)	0.03188 (12)	−0.00524 (8)	−0.00114 (8)	0.00527 (8)
O2W	0.107 (2)	0.0454 (14)	0.0504 (16)	−0.0145 (16)	0.0142 (17)	−0.0025 (14)
O1W	0.076	0.057	0.078	−0.007	−0.017	0.002

### Geometric parameters (Å, °)

**Table:**

C1A—N6A	1.487 (4)	C5A—H51A	0.97
C1A—C2A	1.494 (4)	C5A—H52A	0.97
C1A—H11A	0.97	N3A—H31A	0.9
C1A—H12A	0.97	N3A—H32A	0.9
C1B—N3B^i^	1.487 (4)	N3B—C1B^i^	1.487 (4)
C1B—C2B	1.509 (4)	N3B—H31B	0.9
C1B—H11B	0.97	N3B—H32B	0.9
C1B—H12B	0.97	N6A—H61A	0.9
C2A—N3A	1.489 (4)	N6A—H62A	0.9
C2A—H21A	0.97	Cl1—In1	2.5167 (7)
C2A—H22A	0.97	Cl2—In1	2.5521 (8)
C2B—N3B	1.478 (4)	Cl3—In1	2.5327 (7)
C2B—H21B	0.97	Cl4—In1	2.4959 (7)
C2B—H22B	0.97	Cl5—In1	2.5083 (7)
C4A—N3A	1.492 (4)	Cl6—In1	2.5082 (8)
C4A—C5A	1.498 (4)	O2W—H21W	0.80 (5)
C4A—H41A	0.97	O2W—H22W	0.80 (5)
C4A—H42A	0.97	O1W—H11W	0.84 (5)
C5A—N6A	1.487 (4)	O1W—H12W	0.824 (19)
			
N6A—C1A—C2A	110.3 (2)	C2A—N3A—C4A	111.2 (2)
N6A—C1A—H11A	109.6	C2A—N3A—H31A	109.4
C2A—C1A—H11A	109.6	C4A—N3A—H31A	109.4
N6A—C1A—H12A	109.6	C2A—N3A—H32A	109.4
C2A—C1A—H12A	109.6	C4A—N3A—H32A	109.4
H11A—C1A—H12A	108.1	H31A—N3A—H32A	108
N3B^i^—C1B—C2B	110.3 (2)	C2B—N3B—C1B^i^	111.8 (2)
N3B^i^—C1B—H11B	109.6	C2B—N3B—H31B	109.3
C2B—C1B—H11B	109.6	C1B^i^—N3B—H31B	109.3
N3B^i^—C1B—H12B	109.6	C2B—N3B—H32B	109.3
C2B—C1B—H12B	109.6	C1B^i^—N3B—H32B	109.3
H11B—C1B—H12B	108.1	H31B—N3B—H32B	107.9
N3A—C2A—C1A	111.2 (2)	C1A—N6A—C5A	111.6 (2)
N3A—C2A—H21A	109.4	C1A—N6A—H61A	109.3
C1A—C2A—H21A	109.4	C5A—N6A—H61A	109.3
N3A—C2A—H22A	109.4	C1A—N6A—H62A	109.3
C1A—C2A—H22A	109.4	C5A—N6A—H62A	109.3
H21A—C2A—H22A	108	H61A—N6A—H62A	108
N3B—C2B—C1B	110.3 (2)	Cl4—In1—Cl6	92.66 (3)
N3B—C2B—H21B	109.6	Cl4—In1—Cl5	93.02 (3)
C1B—C2B—H21B	109.6	Cl6—In1—Cl5	88.24 (3)
N3B—C2B—H22B	109.6	Cl4—In1—Cl1	176.49 (2)
C1B—C2B—H22B	109.6	Cl6—In1—Cl1	88.87 (2)
H21B—C2B—H22B	108.1	Cl5—In1—Cl1	90.18 (2)
N3A—C4A—C5A	110.8 (3)	Cl4—In1—Cl3	89.56 (3)
N3A—C4A—H41A	109.5	Cl6—In1—Cl3	176.85 (3)
C5A—C4A—H41A	109.5	Cl5—In1—Cl3	89.41 (3)
N3A—C4A—H42A	109.5	Cl1—In1—Cl3	89.04 (2)
C5A—C4A—H42A	109.5	Cl4—In1—Cl2	87.68 (3)
H41A—C4A—H42A	108.1	Cl6—In1—Cl2	94.98 (3)
N6A—C5A—C4A	110.5 (2)	Cl5—In1—Cl2	176.67 (3)
N6A—C5A—H51A	109.6	Cl1—In1—Cl2	89.04 (2)
C4A—C5A—H51A	109.6	Cl3—In1—Cl2	87.34 (3)
N6A—C5A—H52A	109.6	H21W—O2W—H22W	108 (5)
C4A—C5A—H52A	109.6	H11W—O1W—H12W	109 (5)
H51A—C5A—H52A	108.1		

Symmetry codes: (i) −*x*+1, −*y*+1, −*z*.

### Hydrogen-bond geometry (Å, °)

**Table:**

*D*—H···*A*	*D*—H	H···*A*	*D*···*A*	*D*—H···*A*
O1W—H11W···Cl2	0.84 (5)	2.43 (5)	3.248 (3)	167 (4)
O2W—H21W···Cl6^ii^	0.80 (5)	2.58 (5)	3.353 (3)	163 (5)
O2W—H22W···Cl2^iii^	0.80 (6)	2.37 (6)	3.170 (3)	174 (6)
N3A—H31A···O1W^ii^	0.90	1.91	2.805 (5)	178
N3A—H32A···O2W	0.90	1.95	2.843 (5)	171
N3B—H31B···Cl1^iv^	0.90	2.61	3.233 (3)	127
N3B—H31B···Cl5^iv^	0.90	2.47	3.202 (3)	138
N3B—H32B···Cl1	0.90	2.81	3.273 (3)	113
N3B—H32B···Cl3	0.90	2.37	3.231 (3)	160
N6A—H61A···Cl2^v^	0.90	2.64	3.334 (3)	134
N6A—H61A···Cl3^v^	0.90	2.62	3.330 (3)	136
N6A—H62A···Cl5^vi^	0.90	2.61	3.344 (3)	140
N6A—H62A···Cl6^vi^	0.90	2.77	3.502 (3)	139
C2B—H21B···O1W	0.97	2.47	3.306 (5)	144
C2A—H21A···Cl1^ii^	0.97	2.72	3.470 (3)	135
C2B—H22B···Cl3^i^	0.97	2.83	3.607 (3)	138
C4A—H41A···Cl4^vi^	0.97	2.76	3.620 (3)	148
C4A—H42A···Cl6^iii^	0.97	2.74	3.577 (3)	145

Symmetry codes: (ii) *x*+1, *y*, *z*; (iii) −*x*+1, −*y*+1, −*z*+1; (iv) −*x*, −*y*+1, −*z*; (v) *x*, *y*+1, *z*; (vi) *x*+1, *y*+1, *z*; (i) −*x*+1, −*y*+1, −*z*.
